# Knee Arthrodesis With an Intramedullary Antegrade Rod as a Salvage Procedure for the Chronically Infected Total Knee Arthroplasty

**DOI:** 10.5435/JAAOSGlobal-D-20-00082

**Published:** 2020-09-14

**Authors:** Nicholas M. Brown, Rishi Balkissoon, Bryan M. Saltzman, Bryan Haughom, Jefferson Li, Brett Levine, Scott Sporer

**Affiliations:** From the Department of Orthopaedic Surgery, Loyola University Medical Center, Maywood, IL (Dr. Brown); the Department of Orthopaedic Surgery, University of Rochester Medical Center, Orthopaedics & Rehabilitation, Rochester, NY (Dr. Balkissoon); OrthoCarolina, Sports Medicine Center, Atrium Health Musculoskeletal Institute (MSKI), Charlotte, NC (Dr. Saltzman); Anchorage Fracture & Orthopedic Clinic, Anchorage, AK (Dr. Haughom); UCSF Fresno, Fresno, CA (Dr. Li); the Department of Orthopaedic Surgery, Rush University Medical Center, Chicago, IL (Dr. Levine); and the Department of Orthopaedic Surgery, Cadence Health, Winfield, IL (Dr. Sporer).

## Abstract

Infection is a challenging complication after total knee arthroplasty (TKA) that is often treatable. However, recurrent infection may require resection, amputation, or arthrodesis. The purpose of this study was to evaluate the results of antegrade nailing with an intramedullary rod for the treatment of a chronically infected TKA.

**Methods:**

This study was a retrospective review of a consecutive series of 18 patients with chronically infected TKA treated with arthrodesis using a long antegrade intramedullary nail. There were 11 women and 7 men with an average age of 65 years and average body mass index of 33.8 kg/m^2^. Patients had an average of 7.4 procedures before fusion, and mean follow-up was 50 months. One patient died in the early postoperative period, leaving 17 patients for evaluation. Fusion was defined radiographically as bony bridging of the joint surfaces visible on both anterior-posterior and lateral radiographs. Ambulatory ability, need for chronic antibiotic suppression, complications, and nail removal were recorded.

**Results:**

Sixteen of 17 patients (94%) underwent successful fusion. Ten of 17 patients (59%) continued to ambulate with 9 of these patients requiring an assist device and 7 of 17 patients (41%) predominantly used a wheelchair. Chronic antibiotic suppression was used in 13 of 17 patients (76%). Two patients required nail removal (one for pseudarthrosis and one for possible total hip arthroplasty) and overall 8 of 17 patients (47%) had a complication. Six of 18 patients (33%) died within 2 years of their fusion procedure.

**Discussion:**

Knee arthrodesis with an antegrade intramedullary nail is a viable treatment option for the chronically infected TKA. There was a high rate of successful fusion, along with a high rate of complications, mortality, and need for chronic antibiotic suppression.

**Conclusion:**

Knee arthrodesis with a long IMN is a suitable treatment method as salvage for a chronically infected TKA, but patients should be counseled on the high rate of postoperative complications, poor ambulatory rate, likely need for suppressive antibiotics, and high mortality rate.

Treatment of a chronically infected total knee arthroplasty (TKA) is a difficult clinical problem. Infection occurs in approximately 1% to 2% of primary and 8% to 10% of revision TKA patients.^1,2,3,4^ Treatment options for periprosthetic infection include antibiotic suppression, irrigation and débridement with component retention, one-stage exchange, two-stage exchange, resection arthroplasty, amputation, and knee arthrodesis.^5,6^ Arthrodesis is most commonly required for cases of chronic infection, particularly when the patient is immunocompromised, the infection is polymicrobial, or the organism is particularly virulent or resistant to antibiotics. Arthrodesis is also indicated in cases of significant bone loss, severe ligamentous instability, poor soft-tissue envelope, or deficient extensor mechanism.^7,8,9^

Arthrodesis can be achieved by external fixation, compression plating, and modular or monoblock intramedullary nailing. All of these techniques have advantages and disadvantages, clinical scenarios were each may be preferred, but no one technique is clearly superior.^10,11,12,13,14,15,16,17^ However, intramedullary nailing is a commonly used method because it allows for rigid fixation and early weight-bearing. The downsides of long monoblock nails include the requirement for a second hip incision, potential infectious seeding of the hip, and common mismatch between canal diameters. Modular nailing lacks these drawbacks, but removal is difficult and can be associated with large amounts of bone loss.^7,8,9,14,16,17,18,19^

The senior author's preference is long intramedullary nailing. In this study, a consecutive series of patients who underwent knee fusion as a salvage procedure for chronic infection were reviewed. All patients underwent fusion with the use of a long intramedullary titanium fusion nail. The purpose of this study was to evaluate the results and complications of this fusion technique.

## Methods

**Figure 1:**
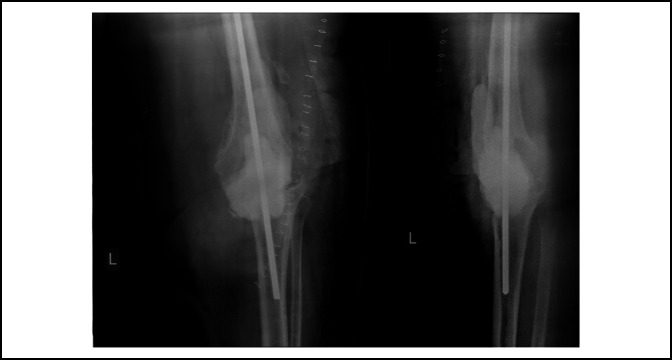
Anteroposterior and lateral radiographs of a knee before arthrodesis.

**Figure 2:**
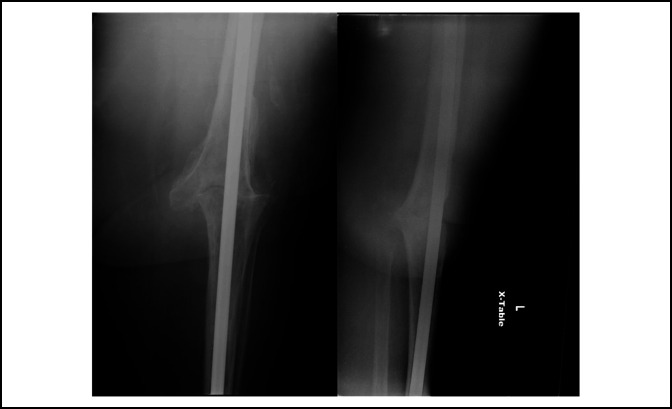
Anteroposterior and lateral radiographs of a knee following arthrodesis with a long intramedullary nail.

After approval from our institutional review board, we retrospectively reviewed a consecutive series of 18 patients with a chronically infected TKA treated by a knee arthrodesis using an antegrade intramedullary nail (IMN) (Figures 1 and 2). These patients were identified by searching the institutional database and the individual surgeons' records. Patients were included in the study if they underwent fusion with a long IMN for the treatment of a chronic periprosthetic joint infection. Patients were excluded if the fusion was performed for other diagnoses or a different technique was used. The chart review was performed by an orthopaedic resident at the senior author's institution.

All patients underwent surgery with the T2 Stryker fusion nail (Kalamazoo, MI). This is a hollow titanium alloy nail that was placed antegrade through the femur with a piriformis starting point. These procedures were performed on a radiolucent table with the patient in slight or “sloppy lateral” position. The intramedullary canals were reamed with flexible reamers after passing a guidewire in either a retrograde or antegrade direction depending on the circumstances of the case. The bone ends were typically already cut relatively congruent for coaptation between them, given the previous knee replacements, and although efforts were made to maintain bone stock, freshening cuts were used as needed to create parallel surfaces. Compression was achieved through manual pressure and the compression feature of the nail was used if more compression was believed necessary. Autograft obtained from patella or femoral condyles was used to help fill any residual space in most cases. No additional antibiotics were added to the construct although antibiotic cement was occasionally used to fill additional space beyond the limits of available autograft. Patients were fused in slight flexion, valgus, and external rotation by using the parallel articular surface cuts. Rivaroxaban (3 patients) or coumadin (14 patients) was used for deep vein thrombosis prophylaxis.

Fusion was defined radiographically as bony bridging of the joint surfaces visible on both anterior-posterior and lateral radiographs. Clinical outcomes were determined by manual chart review of hospital and clinic notes and included ambulatory capacity, need for chronic antibiotic suppression, complications, and nail removal.

## Results

Three surgeons at two institutions done the procedures between December 2004 and June 2013. There were 11 women and 7 men with an average age of 65 years (range 32 to 83 years old) at time of the fusion procedure. These patients had a mean body mass index of 33.8 (range 16.1 to 55.6), mean charlson comorbidity index of 4.3 (range 1 to 9), and had an average of 7.4 (range 5 to 13) previous surgeries on the knee before fusion. All patients had undergone 6 to 8 weeks of treatment with a static antibiotic spacer before fusion. The spacer in all cases consisted of antibiotic cement coated Steinman pins with antibiotic cement filling the remaining joint space. One patient died in the immediate postoperative period of hypotension and anoxic brain injury after a hypotensive episode during cement pressurization. The remaining 17 patients had a mean follow-up of 50 months (range 2 to 150 months). Five of these patients had less than 2-year follow-up because of death within this period.

Sixteen of 17 patients (94%) had a successful radiographic fusion. The patient without a successful fusion required nail removal for chronic infection and pseudarthrosis at 5 months postoperatively. This same patient was subsequently treated with a knee immobilizer and chronic antibiotic suppression. Overall, 13 of 17 patients (76%) were treated with chronic antibiotic suppression. The decision for chronic suppression was based on recommendations by the infectious disease service on a case-by-case basis and was dependent on the infecting organism's susceptibility to oral antibiotics. The most common suppressive oral antibiotics were doxycycline, clindamycin, and cephalosporins. Infecting organisms included polymicrobial (8), methicillin-resistant staph aureus (5), methicillin-sensitive staph aureus (2), coagulase-negative staphylococci (1), and streptococcus (1). Methicillin resistant staph aureus (MRSA) was present in five of eight polymicrobial infections. Ten of 17 patients (59%) continued to ambulate (all but one required an assist device) and 7 of 17 patients (41%) predominantly used a wheelchair.

Overall, 8 of 17 patients (47%) experienced a postoperative complication, with one patient of the original 18 who died in the immediate post-op period. One complication was the previously mentioned patient who had pseudarthrosis secondary to persistent infection that required nail removal. Two of 17 patients (12%) required removal of a symptomatic distal locking screw. Another patient developed postoperative gastrointestinal *Clostridium difficile* infection and was readmitted for sepsis. One patient had their nail removed because they developed ipsilateral hip arthritis and had subsequent plans for total hip arthroplasty. However, cultures were positive at the time of removal with the same infecting organism as the knee (methicillin sensitive staph aureus [MSSA]), and therefore, it was presumed the hip was either seeded at the time of fusion or the nail acted as a conduit from the knee to the hip. The patient had an antibiotic spacer placed because of concern for infection at the time of surgery and eventually underwent permanent hip resection arthroplasty. Another patient required irrigation and débridement at 2 weeks postoperatively for persistent drainage. Additional complications included a patient with a MRSA positive sacral ulcer and another patient who developed a contralateral knee periprosthetic infection with the same infecting organism as their fused knee (MRSA). In addition, 6 patients of the original cohort of 18 died within 2 years of their fusion surgery.

## Discussion

This study retrospectively reviewed 18 patients who had knee fusion done with an antegrade long femoral nail. There was a high rate of fusion (94%), but nearly half of the patients suffered a complication (47%), many lost their ambulatory ability (41%), and the majority continued treatment with chronic oral antibiotics (76%). Furthermore, one-third of patients died within 2 years of their fusion surgery. These results suggest that this is a satisfactory technique for limb salvage of a recurrently infected TKA but associated with high morbidity and mortality.

There are a variety of ways to achieve knee fusion. One method is external fixation, which can be monoplanar, biplanar, or circular. Advantages to this technique include lack of intramedullary infection dissemination, ability to modify alignment, and possibility of simultaneous lengthening. However, disadvantages include pin loosening, pin site infection, difficulty with placement, and delayed weight-bearing.^10,11,12,13,14^ Knee fusion can also be achieved with compression plating. This technique allows for a single incision and immediate compression intraoperatively but often requires extensive soft-tissue stripping, prolonged limited weight-bearing, and knee stabilization.^15^ IMN fixation allows for rigid fixation, early weight-bearing, and seems to have quicker fusion and lower complication rates than the other techniques. Intramedullary fusion is achieved typically with either long monoblock or modular nails.^7,8,9,14,16,17,18,19^

The fusion rate in this study was comparable with other published reports of fusion with an IMN. Overall fusion rates for knee arthrodesis including all techniques range from around 50% to 100%.^13,17,18,19,20,21,22,23^ Leroux et al^21^ demonstrated the exact same fusion rate found in our study showing 16 of 17 patients with chronic infection underwent successful knee fusion with the Stryker T2 nail. Bargiotas et al^22^ demonstrated successful fusion in 10 of 12 patients who underwent treatment with a long IMN because of chronic infection. Puranen et al^24^ showed an 87% fusion rate with the use of a long IMN, although only 15 of 33 patients had the procedure done for a failed arthroplasty (8 infection and 7 aseptic loosening).

Both this report and other similar studies suggest that fusion rates with IMN fixation are higher than with alternate techniques. Hak et al^25^ reported a fusion rate 58% in patients treated with a monoplanar external fixator and 65% in patients treated with biplanar fixators. However, this lower fusion rate may be somewhat because of selection bias because more difficult patients can necessitate fusion with external fixation. Circular fixators have shown higher fusion rates with Manzotti et al^26^ reporting a fusion rate of 100%, and Oostenbroek and Van Roermund^13^ reported a fusion rate of 93% in 15 patients. However, the complication rate in this study was 80%, and patients required treatment for an average of 51 weeks. Schwarzkopf et al^16^ directly compared fusion rates with multiple techniques (IMN, compression plating, and external fixation) in a series of 41 patients, and although the numbers did not reach statistical significance, the patients treated with intramedullary nailing had the highest fusion rates. Mabry et al had similar findings in their study comparing knee fusion with intramedullary nailing to external fixation. This study was underpowered to reach statistical significance, but intramedullary nailing trended toward a higher rate of fusion.^14^

This study found these patients to have a 47% rate of complications, with 76% on chronic antibiotic suppression and 33% dying with 2 years of the procedure. Persistent infection was the etiology of the one pseudarthrosis and the difficulty in definitively clearing the infections was evident, given the high rate of chronic suppression. This is not surprising, given the multiple comorbidities, obesity, advanced age, multiple procedures, and virulent organism profile of the patients in this study. Persistent infection has been reported to occur in 10% to 21% of cases when an IMN is used for fusion.^22,27,28,29^ The rate was much higher in our study likely because all of these patients were fused because of chronic infection. Furthermore, many patients had infections elsewhere in their body, particularly in those infected with staph aureus.

One limitation to this study was its retrospective nature, although this is a fortunately rare procedure, so prospective data collection is difficult. These results were with one technique for the treatment of chronic infection, and although this presents a clear view of the outcomes with this technique and implant, it may limit its applicability to other techniques. In addition, 6 of our 18 patients died within 2 years, which limited the long-term follow-up.

This study found a high rate of fusion in this difficult population of patients with chronic infection after TKA. However, the complication rate was high, as expected in this difficult and unhealthy population of patients. Persistent infection and need for chronic antibiotic suppression were commonly encountered. Polymicrobial and MRSA infection was prevalent. In addition, in one patient utilization of the nail effectively seeded the hip joint precluding subsequent arthroplasty. Overall, we feel that knee arthrodesis with a long IMN is a suitable treatment method as salvage for a chronically infected TKA, but patients should be counseled on the high rate of postoperative complications, poor ambulatory rate, likely need for suppressive antibiotics, and high mortality rate.

## Conclusion

Knee arthrodesis with a long IMN is a suitable treatment method as salvage for a chronically infected TKA, but patients should be counseled on the high rate of postoperative complications, poor ambulatory rate, likely need for suppressive antibiotics, and high mortality rate.
